# Pathophysiologic Mechanisms of Insulin Secretion and Signaling-Related Genes in Etiology of Polycystic Ovary Syndrome

**DOI:** 10.1155/2021/7781823

**Published:** 2021-12-06

**Authors:** Zahra Shaaban, Arezoo Khoradmehr, Amir Amiri-Yekta, Fariborz Nowzari, Mohammad Reza Jafarzadeh Shirazi, Amin Tamadon

**Affiliations:** ^1^Department of Animal Science, College of Agriculture, Shiraz University, Shiraz, Iran; ^2^The Persian Gulf Marine Biotechnology Research Center, The Persian Gulf Biomedical Sciences Research Institute, Bushehr University of Medical Sciences, Bushehr, Iran; ^3^Department of Genetics, Reproductive Biomedicine Research Center, Royan Institute for Reproductive Biomedicine, ACECR, Tehran, Iran

## Abstract

Polycystic ovary syndrome (PCOS) is a common endocrinopathy in women. PCOS is characterized by anovulation, hyperandrogenism, polycystic ovaries, insulin resistance, and obesity. Despite the finding that the genetic origin of PCOS is well demonstrated in previous twin and familial clustering studies, genes and factors that can exactly explain the PCOS pathophysiology are not known. *Objective(s).* In this review, we attempted to identify genes related to secretion and signaling of insulin aspects of PCOS and their physiological functions in order to explain the pathways that are regulated by these genes which can be a prominent function in PCOS predisposition. *Materials and Methods*. For this purpose, published articles and reviews dealing with genetic evaluation of PCOS in women from peer-reviewed journals in PubMed and Google Scholar databases were included in this review. *Results*. The genomic investigations in women of different populations identified many candidate genes and loci that are associated with PCOS. The most important of them are *INSR*, *IRS1-2*, *MTNR1A*, *MTNR1B*, *THADA*, *PPAR-γ2*, *ADIPOQ*, and *CAPN10*. These are mainly associated with metabolic aspects of PCOS. *Conclusions*. In this review, we proposed that each of these genes may interrupt specific physiological pathways by affecting them and contribute to PCOS initiation. It is clear that the role of genes involved in insulin secretion and signaling is more critical than other pathways.

## 1. Introduction

The multigenic multifactorial endocrinopathy that affects about 5–10% of women of the world in their reproductive age is polycystic ovary syndrome (PCOS) [1]. The most important heterogeneous features of PCOS is infertility that originates from anovulation, hyperandrogenism, polycystic ovaries, insulin resistance, obesity, and cardiovascular diseases [2]. But, due to the heterogeneity of PCOS, the exact pathophysiological pathway that initiates the syndrome has not been known yet. Metabolic disorders such as insulin resistance, glucose intolerance, and type 2 diabetes are also observed in PCOS patients [3]. The role of different factors such as genetic, environmental, and developmental origin was explained in PCOS etiology [4]. In many cases, the genes involved in the pathology of these metabolic abnormalities were associated with PCOS and likely with one or more physiological routes interrupted by alteration of these genes. Therefore, the evaluation of these physiological pathways is valuable to clear the etiology of PCOS.

In retrospect to the fact that the etiology of PCOS is not known yet, we should consider two hypotheses hyperinsulinemia and intrauterine environment changes that have been well documented in PCOS formation by animal model studies. They can also be mediated by the genetic background of the individual [5]. Because the reproductive and metabolic implications are mainly observed in the first-class relatives, PCOS is considered as a genetic disorder [6]. Furthermore, familial aggregation studies have confirmed the genetic basis of PCOS hyperandrogenemia (hypersecretion of androgens in PCOS condition) and identified their related susceptible genetic variant in PCOS [4]. Based on twin studies, the heritability of PCOS is approximately 70% [7]. In spite of the effects of the susceptible genetic variants on PCOS that may be influenced by environmental factors, it seems that PCOS develops as a result of a combination of both genetic and environmental agents [4]. As noted, PCOS has heterogeneous characteristics due to involvement of either genetic or environmental factors, although the role of genetic factors is more severe [8]. The strong roles of inheritance and genetic background in PCOS development were confirmed based on twin and familial clustering studies [9, 10]. Unlike the increasing documents proving the heritability of PCOS and the effects of developmental origins of insulin resistance on PCOS development, the exact pathophysiological pathways in etiology of PCOS are not clear [11]. According to genome-wide association studies (GWAS) that identified risk loci for PCOS predisposition, researchers believed that the PCOS inheritance model is more likely to be oligogenic/polygenic than the autosomal dominant [10]. GWAS as a new approach presented a way for unbiased identification of genes without considering the probable role of causative variants [11]. The aim of the present review is brief description of susceptible genes contributed to PCOS development that are related to metabolic pathways such as insulin secretion and signaling. Therefore, according to the gene functions, the involved physiological routes affecting PCOS etiology are explained and novel hypotheses are categorized.

## 2. Materials and Methods

### 2.1. Focused Question

This review was done to answer this question: “What are the roles of insulin secretion and signaling-related genes in pathophysiologic mechanisms of polycystic ovary syndrome?”

### 2.2. Search and Study Selection

Key words and subject terms included (“PCOS” AND “insulin”) OR (“PCOS” AND “insulin” AND “gene”) OR (“PCOS” AND “insulin” AND “signaling”) OR (“PCOS” AND “diabetes”) OR (“PCOS” AND “diabetes” AND “gene”) OR (“PCOS” AND “diabetes” AND “signaling”). The search strategy was applied to PubMed, Elsevier, and Google Scholar databases, focused on the patient-related studies. English language research papers were considered. The review, abstracts without full manuscripts, the manuscripts related to the animal models, or in vitro studies were excluded. Data were collected from the full text of the articles as follows: (i) insulin resistance and diabetes mellitus type 2 or (ii) insulin secretion and signaling and (iii) the obtained results.

On the basis of physiological roles, identified genes related to PCOS can be classified into six groups including the following: a, gonadotropin secretion and actions; b, steroid hormones biosynthesis and functions; c, insulin resistance and type 2 diabetes mellitus; d, insulin secretion and signaling; e, obesity and dyslipidemia; f, chronic inflammatory reactions. In our previous review paper, the roles of genes involved in a, b, d, and f categories were explained in detail [2, 12]. In this review paper, we aimed to clarify the role of effective genes in the insulin secretion and signaling pathway.

## 3. Results and Discussion

### 3.1. Insulin Resistance and Diabetes Mellitus Type 2

Since insulin resistance is one of the underscored phenotypic features of PCOS that can have a genetic source, genetic variants of insulin resistance are also associated with PCOS [13]. Insulin resistance and hyperinsulinemia in adolescents are seen in the early stages of PCOS [14], and adolescents girls with PCOS are exposed to the increased risk of impaired glucose tolerance and diabetes mellitus type 2 [15]. Intrauterine growth retardation (IUGR) leads to alteration in the development of adipose tissue during fetal life [16], while adipose tissue has an effective role in the expansion of insulin resistance in adulthood [17]. Thus, insulin resistance resulting from IUGR can be a source of developmental and preprogramming changes that lead to some abnormalities in adulthood when the growth environment of the fetus is impaired [4]. The point is that PCOS and metabolic syndrome contain some common features such as having an intrinsic origin or due to being out of chronic adult illnesses, they are still originated from developmental age [18].

Obesity is another effective main factor of insulin resistance in PCOS. It is well known that the abdominal phenotype of obesity affects insulin resistance and subsequent compensatory hyperinsulinemia [19]. Obese and nonobese PCOS women have had insulin resistance and pancreatic beta-cell dysfunction, but this situation was not related to glucose intolerance in all PCOS participants [20].

The internal alterations of insulin function and hormonal environment may contribute to the development of PCOS insulin resistance. For instance, the defect of receptor auto-phosphorylation, stimulated by insulin, was exclusively observed in PCOS women and not in other metabolic abnormalities such as obesity, insulin resistance and non-insulin-dependent diabetes mellitus [21]. In some PCOS cases, the auto-phosphorylation of receptors is normal and there is a defect in postbinding receptor events of insulin signaling pathway which lead to insulin resistance [21].

Hyperandrogenism can alter the sensitivity of peripheral tissues to insulin, directly or indirectly, by increasing visceral fat and reducing the secretion of adiponectin. Adiponectin is a main insulin-sensitizing adipokine which contributes to PCOS insulin resistance [6]. Furthermore, there is evidence about heritability of hyperandrogenism and hyperinsulinemia among sisters of PCOS women [22].

**Figure 1:**
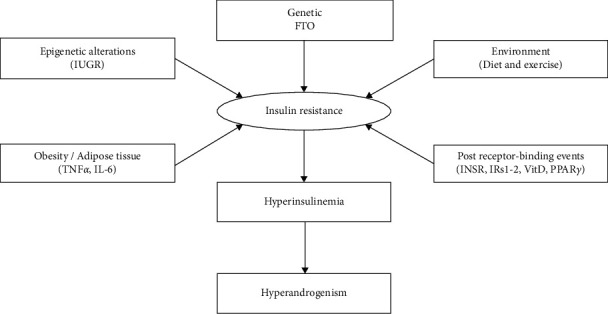
Insulin resistance, producing factors and effective gene in each pathway, and ultimately insulin resistance by the formation of hyperinsulinemia and hyperandrogenism lead to PCOS. Insulin resistance can have different genetic, epigenetic (alteration during intrauterine development), and environmental origins or products derived from the adipose tissue. But, in PCOS condition, insulin resistance is mainly derived from postbinding receptor defects.

Insulin sensitivity is affected by three factors: insulin receptor, peroxisome proliferator-activated receptor-gamma (PPAR-*γ*), and vitamin D [23]. Apa1 polymorphism of vitamin D receptor in Iranian PCOS women is highly associated with this syndrome [24]. A set of pathways leading to insulin resistance are described in Figure 1.

**Figure 2:**
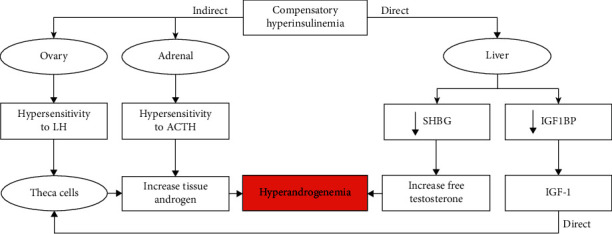
Mechanisms of direct and indirect effects of hyperinsulinemia on hyperandrogenemia. Insulin via increasing the sensitivity of theca cells to LH and adrenal cortex to ACTH elevated the synthesis of androgens in these tissues. Also, insulin via direct effect on the liver and by suppression of production of SHBG and IGF1BP leads to the increased serum level of androgens and eventually hyperandrogenemia.

Insulin resistance leads to compensatory increment of insulin secretion from beta cells resulting in hyperinsulinemia which in turn causes hyperandrogenism. However, when the beta cell is unable to compensate for insulin resistance, hyperglycemia occurs and is followed by glucose intolerance and type 2 diabetes [8]. The reasons of insulin resistance in PCOS are unknown, and it may be the result of postreceptor insulin signaling defects [8]. In addition, several factors, secreted by adipose tissue such as leptin, free fatty acids, interleukin-6, and tumor necrosis factor *α* (TNF*α*), promote insulin resistance and are considered as candidate PCOS genes [25]. The mistake of binding insulin to the receptor or alteration of insulin signal transduction is a forcible mechanism of insulin resistance [26]. The pathophysiological pathway through which hyperinsulinemia leads to hyperandrogenism is explained in Figure 2. Generally, abnormalities of insulin secretion and sensitivity are related to genes involved in insulin signaling and metabolism regulators which are explained in the following.

### 3.2. ADIPOQ

Adiponectin is a protein that is specifically and abundantly expressed in adipocytes. Adiponectin gene polymorphisms affect the levels of this protein, obesity, insulin resistance, and type 2 diabetes [27]. Adiponectin has a special role in modulating insulin sensitivity [28]. Insulin sensitivity is controlled by several genes and interaction of gene products such as adiponectin and resistin (RETN). In a study on PCOS Japanese women, this syndrome was associated with RETN polymorphisms but did not show any association with ADIPOQ gene polymorphisms [29]. Adiponectin gene polymorphisms are more common in PCOS and had a significant correlation with glucose/insulin ratio [27]. In addition, the SNP rs1501299 polymorphism in adiponectin gene, specially based on its role in development of obesity caused by PCOS, was associated with PCOS risk in Chinese Han population [30]. Furthermore, G allele of rs1501299 increased the risk of PCOS in Jordanian population [31]. On the other hand, in a study of Polish women with PCOS, the SNP rs1501299 in the gene ADIPOQ was associated with a reduced risk of disease [32]. A meta-analysis by Liu et al. [33] demonstrated that rs1501299 polymorphisms are significantly associated with PCOS risk in East Asians. But, a meta-analysis of Asian population showed that women with the G276T polymorphism have decreased susceptibility to PCOS [34]. The strong association between 45T/G, +456G15G (T/G), +276 (G/T), 11391G > A, and G276T variants of ADIPOQ and the metabolic features of PCOS, such as insulin resistance, central obesity, dyslipidemia, hypertension, and hyperglycemia, was reported in different populations suggesting that ADIPOQ variants can be considered as the risk factors for PCOS development (Table 1).

### 3.3. CAPN10

Calpain protein is a cysteine protease that plays a role in pro-insulin processing and insulin secretion and action [51]. Women with PCOS are at the risk of impaired glucose tolerance (IGT) or a 2–7-fold increase in type 2 diabetes incidence. Therefore, all genes associated with type 2 diabetes mellitus can play an important role in the pathogenesis of PCOS [8]. CAPN10 gene was the first gene that was identified as type 2 diabetes risk gene [52]. The CAPN10 gene has multiple SNPs. In a meta-analysis study, the association of UCSNP-63 and UCSNP-19 polymorphisms with PCOS was proved [45]. In another meta-analysis study, the role of UCSNP-45 as well as UCSNP-63 and UCSNP-19 polymorphisms was confirmed as the risk factors for PCOS, especially in Asian women [38]. In many of the case-control studies, the association between various polymorphisms of CAPN10 and metabolic traits of PCOS was demonstrated and the diversity of populations was the only difference between studies (Table 1). Accordingly, CAPN10 which plays a role in insulin secretion and pathology of type 2 diabetes can also be an important susceptibility gene for PCOS.

### 3.4. PPAR-*γ*2

PPAR-*γ* is a very important transcription factor which plays a role in regulating glucose homeostasis, lipid metabolism, and ovarian steroidogenesis [53]. The activation of the PPAR-*γ* by the thiazolidinedione drug, used to treat type 2 diabetes, induces differentiation of adipocytes and also increases insulin sensitivity [8]. The PPAR-*γ* gene contains a common SNP Pro12Ala [54]. This gene is expressed primarily in adipose tissue and stimulates the differentiation of preadipocytes into adipocytes, and also, it belongs to the family of nuclear hormone receptors [55]. Pro12Ala polymorphism of PPAR-*γ* gene was proposed in the women of South India as a PCOS susceptibility gene [49]. The meta-analysis has showed that Pro12Ala polymorphism in the PPAR-*γ* has the potential to reduce the risk of polycyclic ovarian syndrome in European patients, which was not observed in the Asians [47]. The Pro12Ala (exon 2) polymorphism of PPAR-*γ* had a protective effect on insulin resistance and beta-cell function in a population of Southern Mediterranean women with PCOS [50]. Therefore, the association between PCOS and Pro12Ala variant of PPAR-*γ* has a racial difference and is mainly related to metabolic abnormalities of PCOS.

### 3.5. Insulin Secretion and Signaling

Hyperinsulinemia is a result of insulin hypersecretion which is caused by the resistance of peripheral tissues to insulin. Also, insulin resistance is mainly due to impaired insulin signaling postbinding receptor pathway [6]. In addition, the glucose homeostasis abnormalities are common in PCOS patients; therefore, in PCOS condition, there is a defect in insulin secretion as well as insulin signaling pathway dysfunction [56]. On the one hand, human epidemiologic studies have demonstrated a correlation between low birth weight and metabolic diseases. On the other hand, IUGR leads to low birth weight which in turn promotes the fetuses into adults with metabolic diseases [57].

**Figure 3:**
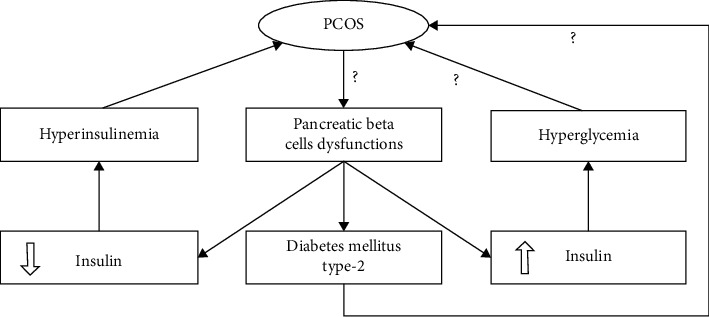
The effect of pancreatic beta-cell dysfunctions on PCOS pathogenesis. Beta-cell dysfunctions lead to type 2 diabetes mellitus and hyperinsulinemia that lead to PCOS by creating hyperandrogenism. However, it is not clear that the PCOS is an introduction to beta-cell abnormalities and then resulted in insulin secretion dysregulation or vice versa.

In PCOS, the initial defect in insulin secretion may indicate the dysfunction of pancreatic beta cells which is related to the occurrence of type 2 diabetes mellitus [58]. There is a basic overlapping link between type 2 diabetes and PCOS [59]. Type 2 diabetes is more likely due to secretion of impaired insulin from pancreatic beta cells. This pathogenic pathway for type 2 diabetes is well known [60], but in previous studies, there is a controversy about the role of beta-cellular impairment in PCOS [59]. The beta-cell dysfunction can affect PCOS development in two ways; firstly, the reduction of beta-cell activity can subsequently cause the impairment of glucose tolerance and hyperglycemia, and secondly, the elevation of beta-cell activity results in hyperinsulinemia, which is followed by adverse effects on peripheral tissues either alone or in combination, and affects the pathogenesis of PCOS [23] (Figure 3).

Women with PCOS have higher levels of fasting insulin and glucose-stimulated insulin, as well as less insulin sensitivity than healthy women who are matched according to age and body mass index [61]. Although the etiology of hyperinsulinemia has not been distinguished yet, clinical and molecular studies have believed that defects in insulin signaling and postbinding receptor, likely due to increase in insulin receptors and insulin receptor substrate-1 phosphorylation, affect metabolic pathways and lead to insulin sensitivity and secretion abnormalities [6]. In addition to pancreatic beta-cell dysfunctions, genes affecting the insulin secretion and signaling also play a role in insulin resistance. Genetic alterations and expression of these genes were investigated and explained in the following sections.

### 3.6. INS

The variable number of tandem repeat (VNTR) polymorphisms in the promoter region of the insulin gene affects its expression [62]. The results of the genetic evaluations of the insulin gene in relation to PCOS are highly controversial. It is mainly due to differences in diagnostic criteria for the identification of affected patients, VNTR genotyping methods, and the racial and geographic background of the participants [8]. Nevertheless, due to the impact of insulin resistance and hyperinsulinemia on anovulation, there may be an association between insulin-related genes and ovulation. While in a meta-analysis study, no association between INS VNTR gene and PCOS was observed [63], INS VNTR class III allele is correlated with increased HOMA-IR and BMI in Kashmiri women with PCOS [64]. A set of previous studies have shown that the variable number of tandem repeat of INS gene is not likely to be dependent on PCOS in different populations (Table 2).

### 3.7. INSR

Insulin receptor gene encodes insulin receptor that plays a pivotal role in insulin signaling pathway, and single nucleotide polymorphisms (SNPs) of this gene are likely to have an effect on PCOS metabolic disorders such as insulin resistance and obesity [13]. In various studies conducted in different populations, there is a strong association between the different varieties of *INSR* gene and PCOS indicating that the INSR, regardless of ethnicity and race, could be a good genetic marker for PCOS (Table 2). The reality is that a C/T polymorphism in the tyrosine kinase domain of INSR gene can be a susceptible variant for PCOS (Table 2). Furthermore, the rs2059807 and rs1799817 in INSR gene were significantly associated with IR in PCOS women in different populations [74, 78–80]. In fact, INSR mediates the effect of insulin resistance on PCOS. But, we should consider findings of studies of insulin resistance in PCOS condition demonstrating that only the metabolic tissues such as liver, skeletal muscle, and fibroblasts are insulin-resistant, whereas the ovary and pituitary tissues remain sensitive to insulin functions [96].

### 3.8. IRS-1 and IRS-2

Recent studies have shown that activation of phosphatidylinositol 3-kinase, being carried out by insulin receptor substrate-1 (IRS-1) and IRS-2 mediators, has an important role in the regulation of insulin-mediated glucose transfer and carbohydrate metabolism [82]. In PCOS women, there is an insulin receptor signaling defect, being accompanied with a decrease in IRS protein, and is related to phosphatidylinositol 3-kinase activity [97]. On the one hand, the Gly972Arg variant of IRS-1 gene was associated with low SHBG levels in adolescent girls with the history of premature pubarche [98]. On the other hand, the relationship between PCOS and insulin resistance is correlated with reduced SHBG-circulating levels leading to increased blood testosterone levels [99]. It is thought that decreasing the tyrosine phosphorylation of IRS-1 and increasing the phosphorylation of IRS-2 Ser312 in PCOS may be initial defects or possible molecular mechanisms in insulin resistance in PCOS [100]. In a meta-analysis, Arg972 polymorphism in IRS-1 has been shown as a PCOS susceptibility allele and it mediates its pathogenesis via an increased level of fasting glucose [81]. In another meta-analysis, the IRS-1 Gly972Arg polymorphism was found to be a risk factor for PCOS susceptibility [82]. The mRNA levels of IRS-1 and IRS-2 were significantly increased as the result of hyperandrogenic environment in PCOS women [101]. However, the value of *IRS-1* and *IRS-2* polymorphisms in association with PCOS is not as the value of INSR gene polymorphisms in PCOS etiology.

### 3.9. THADA

The thyroid adenoma-associated (THADA) gene has been initially identified in chromosomal defects of this genomic region in benign adenoma of thyroid glands, and its intron region was interconnected with peroxisome proliferator-activated receptor-gamma (PPAR-*γ*) [102]. Overtransmission of SNP rs13429458 in THADA suggested that this gene has the capacity to be a new candidate for PCOS [84]. Polymorphisms of THADA may be involved in pathogenesis of both diabetes mellitus type 2 and PCOS [84]. An SNP of THADA, being associated with type 2 diabetes mellitus, indicates that the THADA has the main role in insulin secretion [103]. Thus, further functional genetic studies are required to clarify the exact role of THADA in pathogenesis of both PCOS and diabetes mellitus type 2. However, the SNP rs13429458 of THDAD gene may be a genetic risk factor for PCOS in different populations [76, 87–90].

### 3.10. MTNR1A and MTNR1B

The action of melatonin is mediated by melatonin receptors (MTNRs) which include MTNR1A and MTNR1B, both of which belong to the G-protein coupled-receptors superfamily [92]. MTNR1A is mainly expressed in alpha cells and MTNR1B in beta cells of the pancreas [104]. The MTNR1B gene is a new candidate gene for type 2 diabetes [92], upregulation of which in the pancreatic islets of diabetic patients is a document for the main role of MTNR1B in T2DM pathogenesis [105]. The association of MTNR1B polymorphisms with PCOS has been documented [91, 106]. The rs10830963 SNP MTNR1B was associated with higher insulin resistance and plasma glucose levels and lower beta-cell function in Chinese PCOS women [91]. In another study, it has been demonstrated that the rs2119882 polymorphism of MTNR1A is also associated with metabolic properties of PCOS and could have a causal role in pathogenesis of PCOS [92]. Generally, launching the MTNR1B signaling pathway in the pancreatic beta cells reduces insulin secretion that resulted in elevated fasting glucose levels in PCOS individuals [107]. The MTNR1B rs10830963 and MTNR1B rs2119882 have been involved in the pathophysiology of insulin resistance in the Chinese PCOS women [94] as well as in the meta-analysis of different populations with PCOS [95], which indicates their involvement in the metabolic aspect of PCOS. Therefore, due to having an effective role in the pathology of diabetes, MTNR1A and MTNR1B may be a predisposition factor for metabolic disorders of PCOS.

## 4. Conclusions

The genetic aspect of PCOS is highly supported by different twin and investigations of familial aggregation. According to most of the studies, the critical genes for PCOS development were not reported yet, but scholars are in agreement with INSR, IRS1-2, MTNR1A, MTNR1B, THADA, PPAR-*γ*2, ADIPOQ, and CAPN10 as more susceptible genes in PCOS incidence. The significant point is that these genes were mostly associated with metabolic abnormalities of PCOS. For instance, the role of MTNR1A and MTNR1B, THADA, CAPN10, and PPAR-*γ*2 in pathology of type 2 diabetes and obesity has been confirmed. The animal transgenic model for genes involved in diabetes and insulin resistance can better interpret the physiological pathways involved in the onset of PCOS. New research studies can find the downstream and upstream agents regulating gene transcription and expression by genetic and bioinformatics studies. Then, they can identify most genetic markers which are related to PCOS. Generally, we proposed that after hyperandrogenism, the role of insulin resistance in pathology of PCOS is much more probable. In conclusion, in spite of complexity in finding the root cause, we can claim that PCOS heterogeneity has opened the way for many new research studies.

## Figures and Tables

**Table 1:** Candidate genes involved in etiology of polycystic ovary syndrome related to insulin resistance and type 2 diabetes mellitus.

Gene	Genetic marker(s)	Type of study	Physiologic function	Studied population	Type of polymorphism	References
ADIPOQ, RETN	SNPs at position −420 of the RETN and/or −11377 of the ADIPOQ	Case-control	Insulin resistance and obesity	Japanese	RETN−420G/G	[29]
ADIPOQ	rs1501299, rs2241766, and rs266729	Case-control	Insulin resistance	Jordanian	G allele	[31]
ADIPOQ	rs17300539, rs266729, rs822395, rs822396, rs2241766, rs1501299, rs2241767, rs3774261, and rs17366743	Case-control	Insulin resistance	Saudi Arabian	rs2241766, rs1501299, rs2241767, rs3774261, and rs17366743	[35]
ADIPOQ	SNPs	Case-control	Modulating insulin sensitivity	Minia	Higher genotyping distributions of TG, GG, and TT	[27]
ADIPOQ	T45G and G276T	Meta-analysis	Insulin resistance, obesity, and T2DM	Asian	G276T	[34]
ADIPOQ	rs1501299	Case-control	Lipid profile	Polish	GG	[32]
CAPN10	UCSNP-44, UCSNP-43, UCSNP-19, and UCSNP-63	Case-control	Ca-mediated intracellular signaling, and insulin secretion	Spanish	UCSNP-44	[36]
CAPN10	CAPN10 haplotypes	Haplotype-phenotype correlation	Ca-mediated intracellular signaling, and insulin secretion	Spanish	UCSNP-44	[37]
CAPN10	SNPs	Meta-analysis, meta-regression	Ca-mediated intracellular signaling, and insulin secretion	Asian	UCSNP-19, UCSNP-63, and UCSNP-45	[38]
CAPN10	UCSNP-43, UCSNP-44, UCSNP-19, and UCSNP-63	Cross-sectional population-based	Ca-mediated intracellular signaling, and insulin secretion	Spanish	UCSNP-44, UCSNP-43, and UCSNP-19	[39]
CAPN10	UCSNP-43, UCSNP-19, and UCSNP-63	Case-control	Ca-mediated intracellular signaling, and insulin secretion	Chilean	UCSNP-43	[40]
CAPN10	UCSNP-43, UCSNP-19, and UCSNP-63	Cross-sectional	Ca-mediated intracellular signaling, and insulin secretion	Brazilian	UCSNP-43	[41]
CAPN10	UCSNP-44, UCSNP-43, UCSNP-56, UCSNP-19, and UCSNP-63	Case-control	Ca-mediated intracellular signaling, and insulin secretion	Indian	UCSNP-44	[42]
CAPN10	UCSNP-43 and rs3792267	Case-control	Ca-mediated intracellular signaling, and insulin secretion	Greek	UCSNP-43	[43]
CAPN10	UCSNP-43 and rs3792267	Case-control	Ca-mediated intracellular signaling, and insulin secretion	Indian	ND	[44]
CAPN10	UCSNP-19, UCSNP-63, UCSNP-44, and UCSNP-43	Meta-analysis	Ca-mediated intracellular signaling, and insulin secretion	Different populations	UCSNP-19 and UCSNP-63	[45]
PPAR-*γ*	Gly482Ser, PPAR-*α* Leu162Val, PPAR-*δ* rs2267668A/G, PPAR-*δ*−87T/C, PPAR-*γ*2 Pro12Ala, and PPAR-*γ*2-−681C/G	Case-control, meta-analysis	Glucose homeostasis, lipid metabolism, transport, and storage	Caucasian	Gly482Ser and Pro12Ala	[46]
PPAR-*γ*	Pro12Ala	Meta-analyses	Glucose homeostasis, lipid metabolism, transport, and storage	Different population	Pro12Ala	[47]
PPAR-*γ*2	Pro12Ala	Case-control	Glucose homeostasis, lipid metabolism, transport, and storage	Chinese	ND	[48]
PPAR-*γ*2	Pro12Ala	Case-control	Glucose homoeostasis, lipid metabolism, and adipocyte differentiation	South Indian	Pro12Ala	[49]
PPAR-*γ*	Pro12Ala (exon 2) and His447His (exon 6)	Case-control	Insulin resistance and adiposity	Southern Mediterranean	Pro12Ala (exon 2)	[50]

Abbreviations: ADIPOQ, adiponectin; CAPN10, calpain 10; PPAR-*γ*, peroxisome proliferator-activated receptor-gamma; ND, no data.

**Table 2:** Candidate genes involved in the etiology of polycystic ovary syndrome related to insulin secretion and signaling.

Gene	Genetic marker(s)	Type of study	Physiologic function	Studied population	Type of polymorphism	References
INS	INS VNTR	Case-control	Insulin secretion	Czech	ND	[65]
INS	INS VNTR	Case-control, family-based association, quantitative trait analyses	Insulin secretion	British/Irish	ND	[66]
INS	−23/Hph I	Case-control	Insulin secretion	Korean	ND	[67]
INS	INS VNTR	Meta-analysis	Insulin secretion	Different	ND	[63]
INS	INS VNTR	Case-control	HOMA-IR	Kashmiri	ND	[64]
INSR	C/T SNP	Case-control	Insulin signaling	American	Exon 17C/T SNP	[68]
INSR	D19S884	Case-control	Insulin signaling	Caucasian	D19S884	[69]
INSR	T/C SNP	Case-control	Insulin signaling	Chinese	T/C SNP	[70]
INSR	Nine SNPs	Case-control	Insulin resistance	Korean	+176477C > T	[13]
INSR	Exon 17 C/T	Case-control	Insulin signaling	Turkish	ND	[71]
INSR	C/T SNP at exon 17	Case-control	Insulin signaling	Chinese	C/T SNP at exon 17	[72]
INSR	C/T polymorphism	Case-control	Insulin signaling	Indian	C/T polymorphism	[73]
INSR	rs1799817, rs2059807, rs8108622, and rs10500204	Family association study	Insulin signaling	Chinese Han	ND	[74]
INSR	rs3786681, rs17253937, and rs2252673	Family-based analysis	Insulin signaling	Chinese Han	rs2252673	[75]
INSR	Susceptibility loci	Case-control	Insulin signaling	Europeans	INSR	[76]
INSR	Genotype and allele frequencies	Case-control	Insulin signaling	Indonesian	ND	[77]
INSR	rs1799817	Case-control	Insulin signaling	Saudi Arabian	Allele T	[78]
INSR	rs2059807 and rs1799817	Case-control	Insulin signaling	Indian	rs2059807 and rs1799817	[79]
INSR	rs2059807	GWAS	Metabolic syndrome and insulin resistance	Han Chinese	rs2059807	[80]
INSR	INSR mutation	Case report	Insulin signaling	Jamaican	p.His1157Gln	[3]
ΙNSR, IRS-1, and IRS-2	Gly972Arg (G972R)	Meta-analysis	Insulin signaling	Different	Gly972Arg (G972R) variant in IRSs	[81]
IRS-1 and IRS-2	Gly972Arg and Gly1057Asp	Meta-analysis	Insulin signaling	Different	Gly972Arg 1	[82]
IRS-2	295 SNPs	Case-control	Insulin signaling	Caucasian	Three SNPs	[83]
THADA	rs13429458	Case-control	Regulation of energy homeostasis	Chinese Hui	ND	[76]
THADA	2p21 chr	Case-control	Regulation of energy homeostasis	European	THADA	[83]
THADA	Susceptibility loci	Case-control	Regulation of energy homeostasis	Europeans	THADA	[76]
THADA	rs13429458, rs12478601, rs13405728, rs10818854, and rs2479106	Family-based analysis	Regulation of energy homeostasis	Chinese Han	rs13429458	[84]
THADA	rs12478601	Case-control	Pancreatic beta-cell function	Iraqi	ND	[85]
THADA	rs13429458	GWAS	Insulin resistance	Han Chinese	rs13429458	[80]
THADA	rs13429458	GWAS	Glucose metabolism	Indian	ND	[86]
THADA	rs13429458	Meta-analysis	Regulation of energy homeostasis	Asian	Minor allele (C)	[87]
THADA	rsl3429458	Case-control	Glucose metabolism	Xinjiang Uygur	Minor allele (T)	[88]
THADA	rs13429458	Meta-analysis	Glucose metabolism	Chinese	rs13429458	[89]
THADA	rs13429458	Case-control	Glucose metabolism	Indian	rs13429458	[90]
MTNR1B	rs10830963 and rs10830962	Case-control	Regulator of circadian rhythms and reproductive processes	Chinese Han	rs10830963	[91]
MTNR1A	rs2119882	Case-control	Regulator of circadian rhythms and reproductive processes	Chinese Han	rs2119882	[92]
MTNR	rs2119882 and rs10830963	Family association study	Regulator of circadian rhythms and reproductive processes	Chinese Han	rs2119882	[93]
MTNR1A MTNR1B	rs2119882 and rs10830963	GWAS	Glycolipid metabolism	Chinese	MTNR1A rs2119882 and MTNR1B rs10830963	[94]
MTNR1A MTNR1B	rs2119882 and rs10830963	Meta-analysis	Insulin resistance	Different populations	MTNR1B rs10830963 and MTNR1B rs2119882	[95]

Abbreviations: GWAS, genome-wide association study; HOMA-IR, homeostatic model assessment for insulin resistance; INS, insulin gene; INSR, insulin receptor; IRS, insulin receptor substrate; MTNR1A, melatonin receptor 1A; THADA, thyroid adenoma associated; ND, no data.

## Data Availability

No data were used to support this study.
